# Ipsilateral synchronous papillary renal neoplasm with reverse polarity and urothelial carcinoma in a renal transplant recipient: a rare case report with molecular analysis and literature review

**DOI:** 10.1186/s13000-023-01405-w

**Published:** 2023-11-03

**Authors:** Daosheng Li, Fenfen Liu, Yiqian Chen, Ping Li, Yuyu Liu, Yu Pang

**Affiliations:** 1 Department of Pathology, the Affiliated Tai’an City Central Hospital of Qingdao University, Tai’an, 271000 China; 2 Department of Urology, the Affiliated Tai’an City Central Hospital of Qingdao University, Tai’an, 271000, China; 3 Department of Rehabilitation, the Affiliated Tai’an City Central Hospital of Qingdao University, Tai’an, 271000 China; 4 Department of Hematology, the Affiliated Tai’an City Central Hospital of Qingdao University, Tai’an, 271000, China

**Keywords:** Papillary renal neoplasm with reverse polarity, Urothelial carcinoma, Renal transplantation, KRAS mutation, FGFR3 mutation, KDM6A mutation

## Abstract

**Background:**

Renal transplant recipients (RTRs) have a 3- to 5-fold higher risk of developing malignant tumors than the general population, with new malignant tumors after transplantation considered to be the leading cause of death in RTRs. In pathological practice, it is rare for neoplasms with different histology to be located in the same organ. We report the first case of a synchronous papillary renal neoplasm with reverse polarity (PRNRP) and urothelial carcinoma (UC) in the ipsilateral kidney in an RTR. Molecular detection was conducted by next-generation sequencing.

**Case presentation:**

A 68-year-old female suffered from uremia 19 years ago and underwent renal transplantation (RT) after receiving dialysis for 6 months. Hematuria occurred one month ago and an enhanced CT showed that there were two abnormal density foci in the middle and lower parts of the autologous left kidney. A laparoscopic left nephrectomy and ureterectomy were performed. Gross examination revealed a mass (I) in the left renal parenchyma, 2*1.8*1.5 cm in size, that protruded from the renal capsule, and a cauliflower-like mass (II), 5*2.5*2 cm in size, adjacent to the mass (I). Microscopic findings revealed these lesions were PRNRP and UC, respectively. PCR analysis revealed a KRAS gene mutation (G12D in exon 2) in the PRNRP, while NGS analysis revealed FGFR3 (S249C in exon 7) and KDM6A (Q271Ter in exon 10 and A782Lfs in exon 17) mutations in the UC.

**Conclusions:**

We report here for the first time an extraordinarily rare case of synchronous renal tumors of a PRNRP and UC in the ipsilateral kidney of an RTR. We identified simultaneous KRAS, FGFR3, and KDM6A mutations in two different renal masses in the ipsilateral kidney. Pathologic assessment with comparative molecular analysis of mutational profiles facilitates tumor studies after RT and may be of great value in clinical management strategies.

## Background

Post-transplant malignant tumors have become the third leading cause of death in renal transplant recipients (RTRs) [1, 2]. Compared with the general population, the overall incidence of malignant tumors in RTRs is 3–5 times higher [3], of which lymphoma and skin cancer are the most common malignant tumors, followed by urogenital neoplasms [4, 5]. The risk of renal and bladder cancers in RTRs is three times higher than that in the general population [6–8]. Urothelial carcinoma (UC) is reported to be more common in Chinese patients after transplantation and is also the main cancer affecting dialysis patients. UC in RTRs is more common in the upper urinary tract than in the bladder, although in dialysis patients this occurrence is exactly opposite [9]. In addition, cancers in RTRs have faster growth rates and invasiveness and tend to be multiple, spreading locally and early in the whole body [10].

In this paper, we report the simultaneous occurrence of a papillary renal neoplasm with reverse polarity (PRNRP) and UC in the autologous left kidney after renal transplantation (RT). In 2019, Al-Obaidy et al. [11] first described PRNRP as a rare inert tumor with unique morphological and immunohistochemical features associated with a KRAS mutation. A subsequent paper by Al-Obaidy et al. [12] also described bilateral synchronous renal malignant tumors and concurrent benign and malignant tumors in the ipsilateral kidney. That paper described a review of 26 cases of 50 PRNRP that had renal tumors with different histological subtypes in the ipsilateral kidney, including PRCCs, clear cell RCCs, acquired cystic disease-associated RCCs, chromophobe RCCs and oncocytomas. Shen et al. [13] also reported a case of PRNRP and a clear cell papillary RCC. However, a PRNRP and UC after RT have not been reported in an ipsilateral kidney in the literature. In this paper, we describe for the first time a case of synchronous PRNRP and UC in the ipsilateral kidney after RT investigated by immunohistochemistry (IHC), polymerase chain reaction (PCR) analysis, and next-generation sequencing (NGS).

## Case presentation

**Fig. 1:**
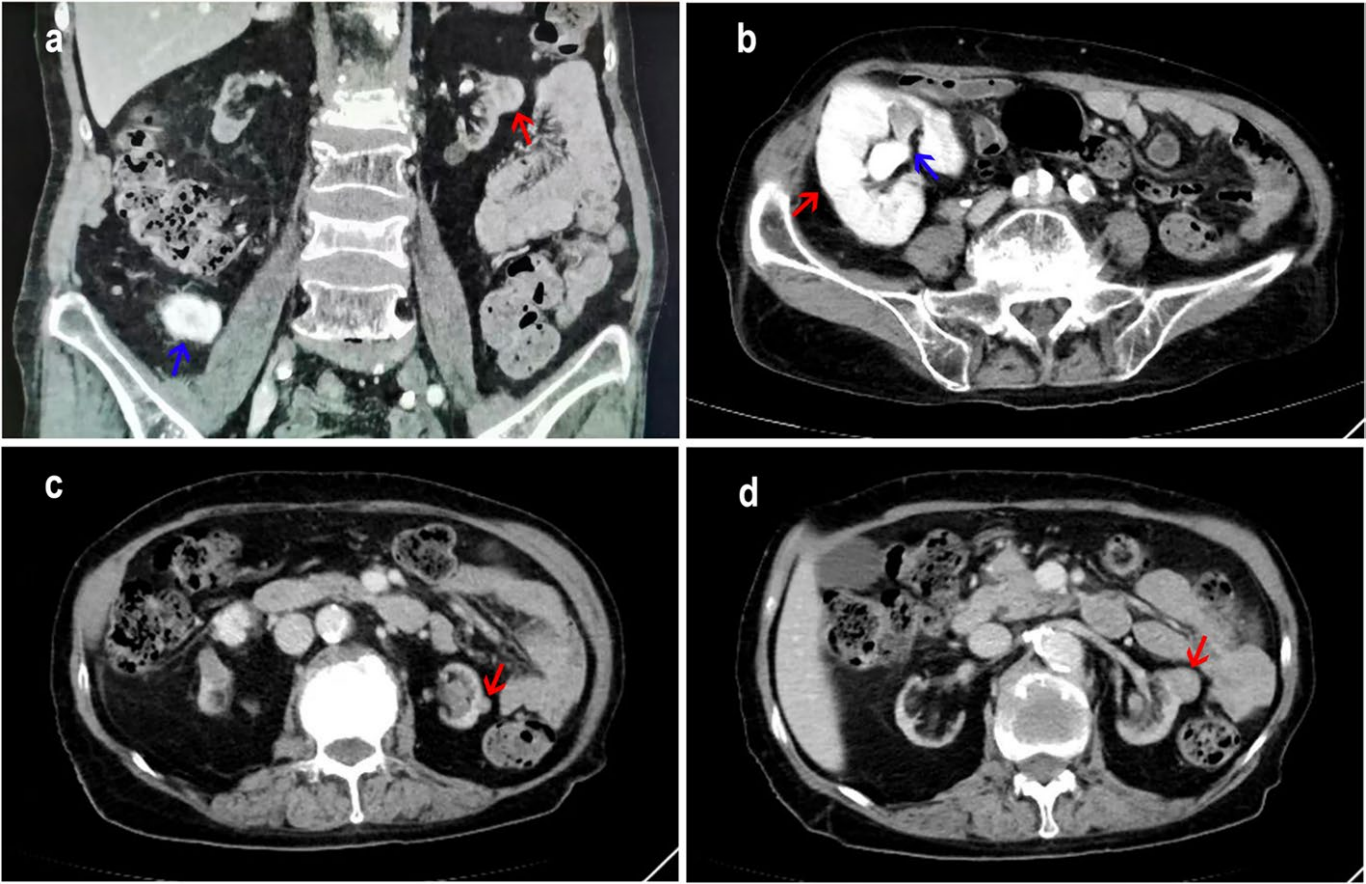
Enhanced CT findings in the patient. **a** The transplanted kidney shadow in the right iliac fossa (blue arrow). A slightly low-density shadow in the autologous left kidney, with some protrusion beyond the kidney contour (red arrow). **b** The transplanted kidney in the right iliac fossa (red arrow), with dilatation of the renal pelvis and some renal calyces which also contained an effusion (blue arrow). **c** Small rounded, slightly low-density shadows (mass (I)) in the autologous left kidney (red arrow). **d** Large rounded, slightly low-density shadows (mass (II)) in the autologous left kidney (red arrow)

**Fig. 2:**
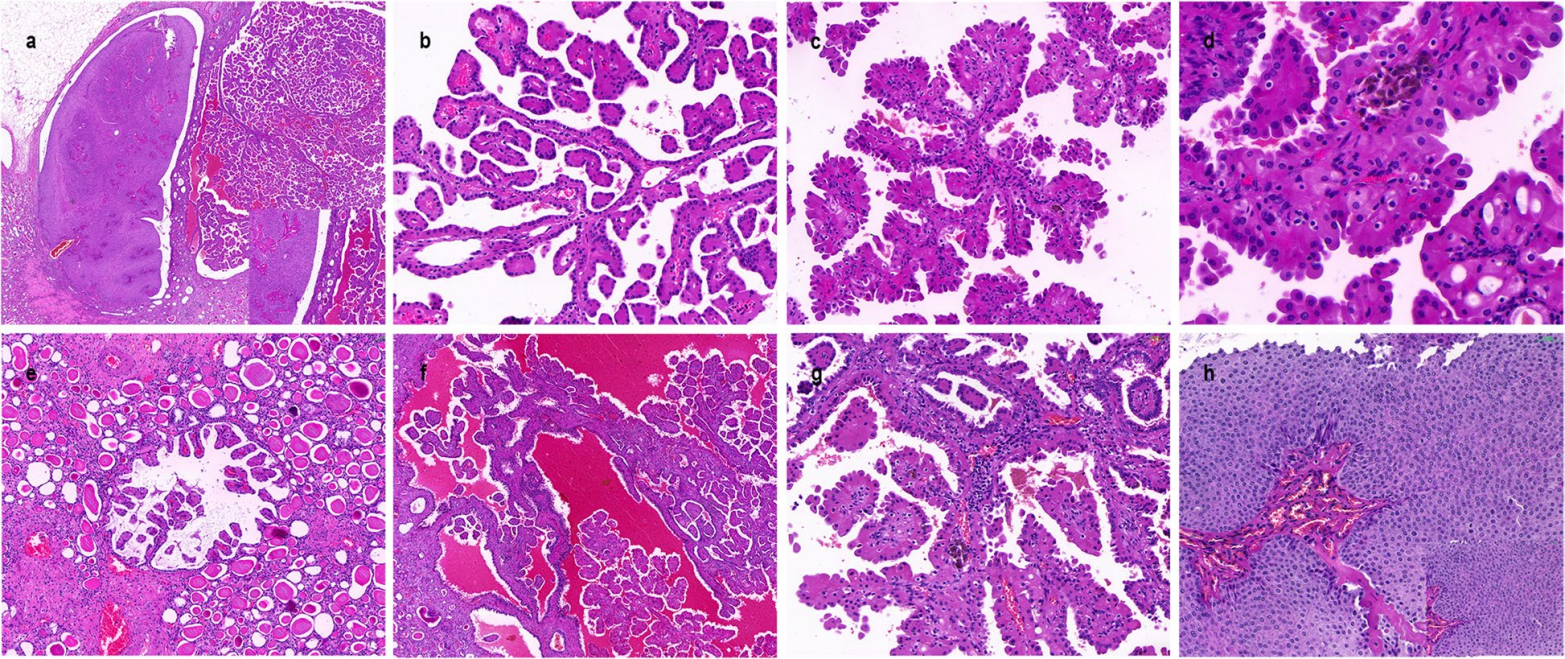
Hematoxylin–eosin (HE) staining of papillary renal neoplasm with reverse polarity (PRNRP) and urothelial carcinoma (UC). **a** PRNRP coexisting with an adjacent UC (HE × 20, HE × 100). **b** Branching papilla with inverted low-grade nucleus of PRNRP (HE × 200). **c** Eosinophilic ‘hobnail cells’ in the PRNRP (HE × 200). **d** Intracytoplasmic vacuoles and hemosiderin deposition in the PRNRP (HE × 400). **e** Small focal invasions into the renal parenchyma in the PRNRP (HE × 100). **f** Hemorrhage can be seen in the local interstitium of PRNRP. (HE × 50). **g** A small amount of lymphocyte infiltration locally in the interstitium of the PRNRP. (HE × 200). **h** UC showed papillary growth, an increased number of cell layers, disordered polarity, mild atypia of the cells with an occasional mitotic division (HE × 200, HE × 400)

**Fig. 3:**
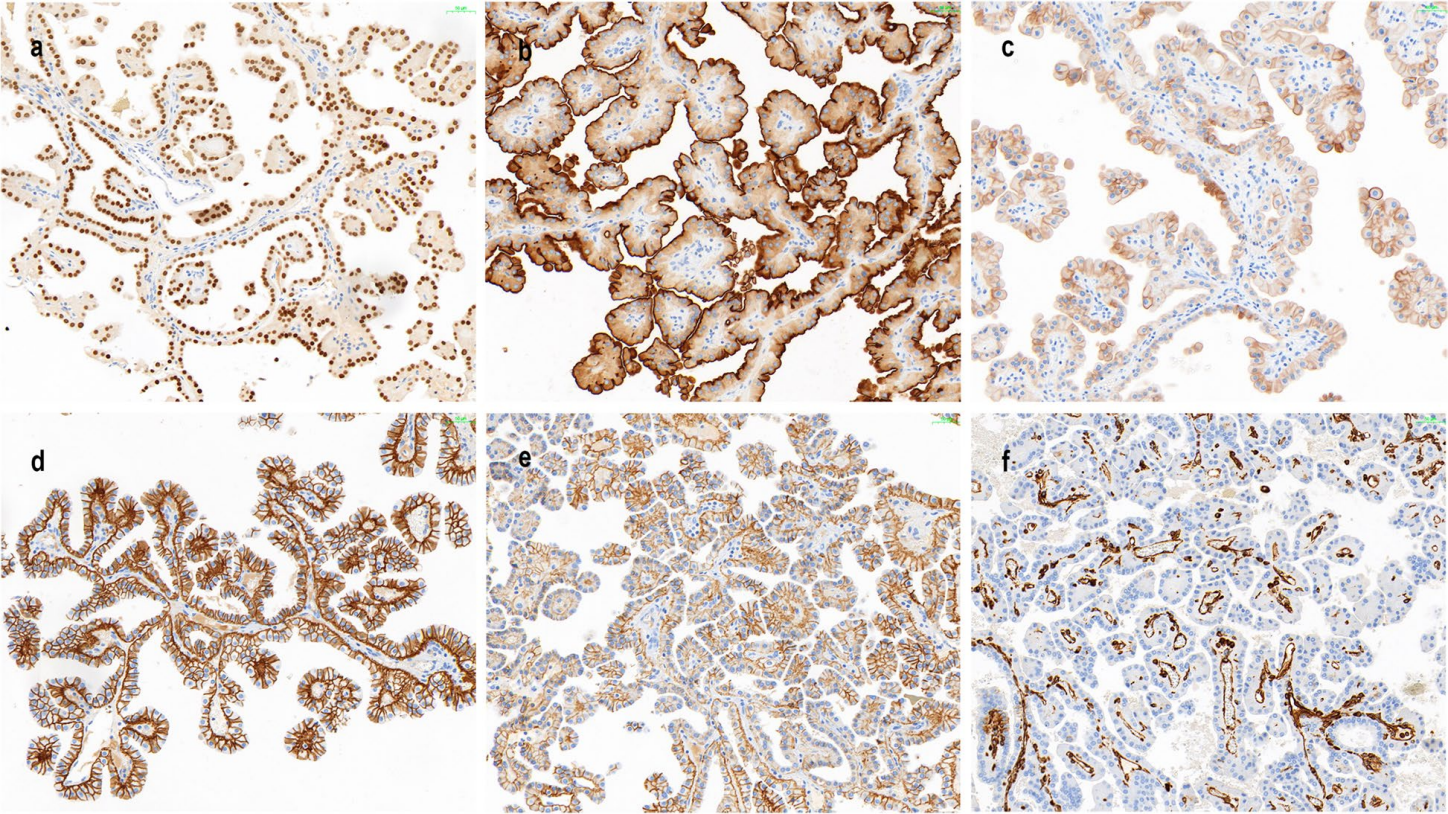
Immunohistochemical profiles of papillary renal neoplasm with reverse polarity (PRNRP). **a** GATA3 showing strong nuclear positivity (IHC × 200). **b** MUC1 was strongly positive on apical membrane, exhibiting polar reversal (IHC × 200). **c** 34bE12 showing membrane positivity (IHC × 200). **d** Basolateral-membranous ‘cup-like’ staining of E-cadherin (IHC × 200). **e** Basolateral-membranous ‘cup-like’ staining of Ksp-cadherin (IHC × 200). **f** Vimentin was negative in tumor cells but positive in the fibrovascular axis (IHC × 200)

In 2004, a 68-year-old female received an RT after six months of dialysis for uremia. After RT, the patient has been using immunosuppressants, and the regimen is: cyclosporin A(CsA) + mycophenolate mofetil (MMF) + prednisone(Pred). No rejection was observed after transplantation and the function of the transplanted kidney was good. In 2022, the patient developed hematuria. An enhanced CT showed a shadow in the transplanted kidney in the right iliac fossa and that the renal pelvis and some renal calyces were dilated and contained an effusion (Fig. 1a and b). The volume of both autologous kidneys had become smaller and the cortex and medulla had become thinner. Two round, slightly low-density shadows were seen in the middle and lower parts of the autologous left kidney, some of which protruded beyond the contour of the kidney, with unclear boundaries (Fig. 1c and d). No abnormalities were found in the bladder. A laparoscopic resection of the left kidney and ureter was performed. Gross pathological examination showed a soft and friable mass (I) in the left renal parenchyma, 2*1.8*1.5 cm in size, with a grayish-red section, thin papillary, that was clear from the surrounding boundary and protruded from the renal capsule. A cauliflower-like mass (II), 5*2.5*2 cm in size, was seen in the renal pelvis adjacent to mass (I), with a grayish-red section, soft and friable texture, and unclear boundary from the surrounding area. Microscopically, mass (I) was adjacent to mass (II) (Fig. 2a). Mass (I) had a clear boundary from the surrounding tissues and was formed mainly by complex branching papillary structures with a fibrovascular axis (Fig. 2b). The papillae were covered with a single layer of cubic cells, with abundant cytoplasm and eosinophilia. The nuclei were round and located at the top of the cell away from the nuclear basement membrane, and were characterized by "reverse polarity", with no obvious nucleolus. The nuclei were classified as grade 1 according to the WHO/ISUP classification system. Eosinophilic hobnail cells, intracytoplasmic vacuoles, and hemosiderin deposition could be seen locally in mass (I) (Fig. 2c and d). Mass (I) exhibited small focal invasions into the renal parenchyma, and a hemorrhage and a small amount of lymphocyte infiltration in the local interstitium (Fig. 2e-g). Mass (II) showed papillary growth, local papillae that merged with each other, an increased number of cell layers, disordered polarity, and mild atypia of the cells with occasional mitotic division (Fig. 2h). We performed IHC staining that showed the tumor cells in mass (I) diffusely and strongly expressed GATA3, MUC1, EMA, E-cadherin, 34bE12, Ksp-cadherin, CK7, and PAX-8 (Fig. 3a-e), while CD117, CAIX, CK20, RCC, and TFE3 were negative. Vimentin, CD10, and P504s were locally expressed in different degrees, and the positive index of Ki67 was about 1% (Fig. 3f). Mass (I) was diagnosed as a PRNRP with a pathologic stage of pT1a N0 M0, and histological grade (WHO/ISUP nuclear grade) of 1. Because microsatellite instability (MSI) is common in upper urinary tract UC (UTUC) [14], the staining IHC for MLH1, MLH2, PMS2, and MSH6 in mass (II) were all positive, while HER2 was negative. Mass (II) was diagnosed as an invasive low-grade UC with a pathological stage of pT1b N0 M0. In August of last year, cystoscopy revealed seven masses in the anterior, bottom, and lateral wall of the bladder and the left ureter opening. The maximum diameter of the masses was about four mm, and they had a pedicle. Two regions were biopsied with, and the samples were then sent for pathology, which showed a papillary urothelial neoplasm with low malignant potential. This was followed by infusion chemotherapy, and part of the left ureter was resected in July of this year. Postoperative pathology confirmed non-invasive low-grade UC. The patient was followed up until September 1, 2023, and the patient was still alive.

**Fig. 4:**
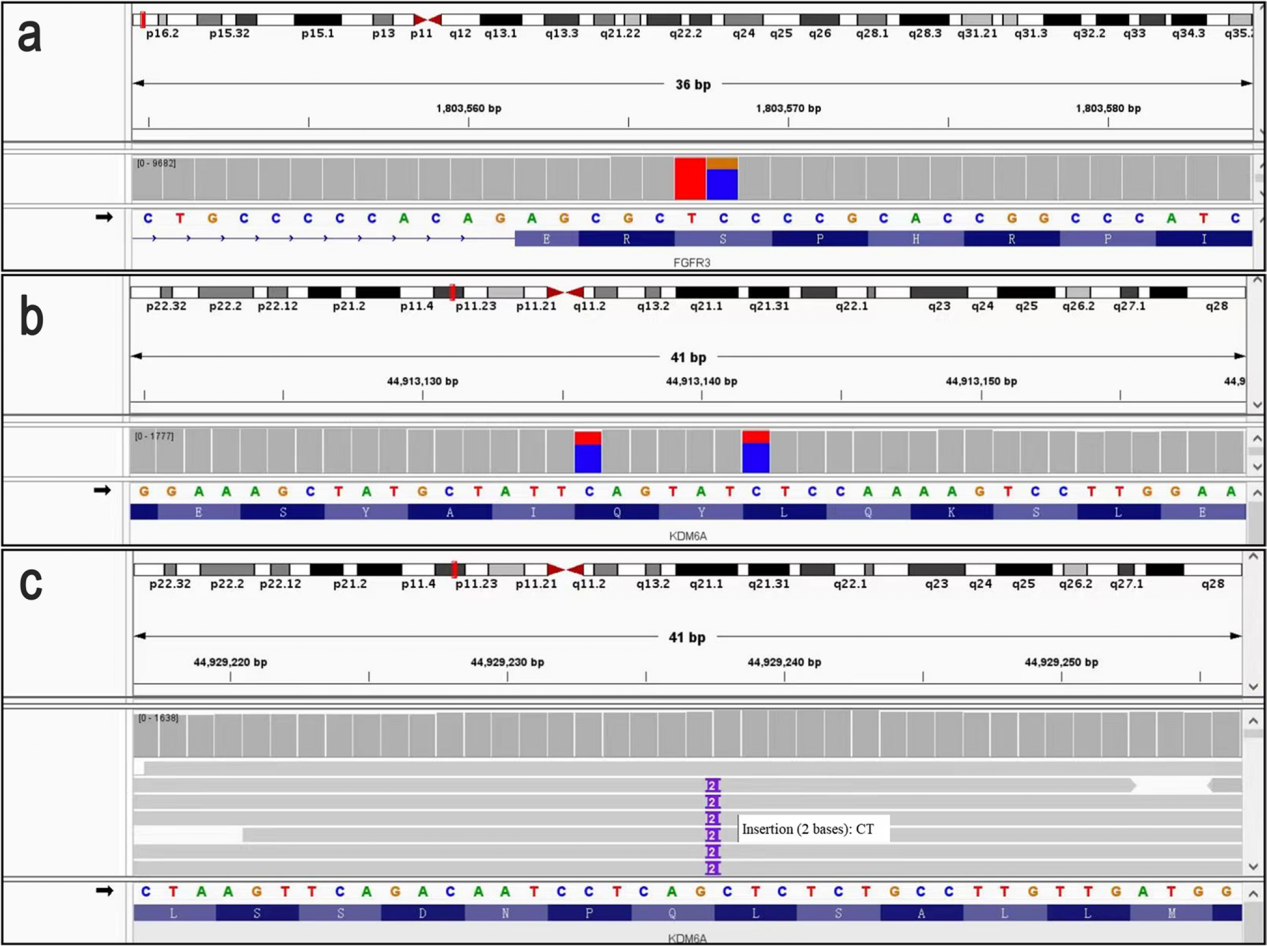
NGS analysis revealed FGFR3 and KDM6A mutations in the urothelial carcinoma (UC). **a** FGFR3 (S249C in exon 7) mutation. **b** KDM6A( Q271Ter in exon 10) mutation. **c** KDM6A (A782Lfs in exon 17) mutation

PCR analysis revealed a KRAS (G12D in exon 2) mutation in the PRNRP, while NGS analysis showed FGFR3 (S249C in exon 7), KDM6A (Q271Ter in exon 10 and A782Lfs in exon 17) mutations in the UC (Fig. 4).

## Discussion

Tumors with different histological types are rarely seen pathologically in the same organ and are even rarer in RTRs. We report for the first time an RTR with a KRAS-mutated PRNRP and an FGFR3 and KDM6A mutated UC in the ipsilateral kidney. Between 5–6% of multiple ipsilateral renal tumors develop a contralateral metachronous recurrence, with the risk five times that of patients with a sporadic single tumor [15, 16]. Post-transplant tumor management depends on the type and severity of the tumor, with different tumors having different invasiveness, recurrence rates, and prognosis. Treatment options therefore vary, especially when planning for nephron-sparing surgery and active surveillance for some renal masses. For multiple renal masses, close attention should be paid to the different histological subtypes of each mass, and the prognosis of each mass should be assessed separately. Therefore, it is necessary to further understand the type, prognosis, and molecular characteristics of urological tumors in RTRs, in order to customize an appropriate therapeutic regimen for each case.

In 2019, Al-Obaidy and his colleagues [11] reported 18 cases of papillary renal neoplasm with unique morphological characteristics and named it "PRNRP". Because PRNRP is rare and only recently defined, it is currently poorly studied and has not been reported in RT patients. This renal tumor exhibits inert biological behavior with no recurrence, metastasis, or tumor-related death, making it important to distinguish PRNRP from other renal cell carcinomas with papillary architecture and eosinophilic cytoplasm. The main morphological characteristics of PRNRP are usually observed as a well-circumscribed and encapsulated lesion with branching papillae of slender fibrovascular axes or tubular papillary structures; a surface that is covered with a monolayer of cubic or columnar cells; abundant eosinophilic cytoplasm; rounded nuclei arranged in the cavity surface away from the basement membrane, "reverse polarity " characteristics with inconspicuous nucleoli with a low WHO/ISUP nuclear grade; rarely mitotic; and accompanied by hemorrhage and cystic degeneration, but no psammoma. Minor morphological features include edematous and hyalinized papillae, filled with transparent to eosinophilic liquid in which phagocytes can be observed; eosinophilic hobnail cells; intracytoplasmic vacuoles; a peritumoral lymphoid cuff or a small amount of lymphocyte infiltration; foamy histiocyte aggregation; intracellular hemosiderin; and mast cell infiltration in the stroma. These morphologies are typically focal and only present in a small subset of cases [12, 17]. A recent study by Yang [18] found that among 11 cases of PRNRP, 5 cases had multifocal or patchy necrosis, 6 cases had a small focal invasion of renal parenchyma or a pseudo capsule, and 1 case had renal capsule breakthrough with neural invasion. Although the morphology of these patients showed an invasive growth pattern, all the patients were alive without metastasis or recurrence at the end of the follow-up period. In our case, we also found a small focal invasion of the renal parenchyma. Immunophenotypically, tumor cells usually express EMA, CK7, GATA3, and L1CAM, while CD117 and CAIX are negative. EMA is a recognized molecule that displays cell polarity, with its apical membrane showing enhanced immunostaining and exhibiting polar reversal. However, our experience suggests that immunostaining of MUC1 is better at exhibiting polar reversal than EMA. GATA3 and L1CAM always exhibit diffuse and strong expression, with a recent study of PRNRP showing that GATA3 and L1CAM demonstrated more heterogeneous staining in a pattern of varied intensity (weak to strong) and extent (20% to 100% of the tumor cells) [19]. In our case, diffuse and strong expression of GATA3 was observed. Because our laboratory does not perform L1CAM, as an alternative we performed immunostaining for E-cadherin and Ksp-cadherin and found it to be expressed diffusely in the basolateral membrane, while 34bE12 was diffusely and strongly positive. These findings were consistent with a recent report that positive expressions of E-cadherin and 34bE12 are found in 87.5% (14/16) and 75% (12/16) of PRNRP cases, respectively [13, 20]. The co-expression of GATA3 and 34bE12 is relatively rare in renal cell tumors but is often seen in tumors of distal tubule or collecting duct origin, such as a collecting duct carcinoma [21, 22]and clear cell papillary RCC [23, 24]. The co-expression of GATA3 and 34bE12 in PRNRP may also point to its distal tubule or collecting duct origin. Proximal renal tubular markers such as vimentin, CD10, CD15, and AMACR may be positive in PRNRP, but are usually weak and focal. PRNRP has a high frequency of KRAS missense mutations, with KRAS mutations found in 57-93% of PRNRPs at a total frequency of 85%, with the most common KRAS mutation being p.G12V (54%) [12, 17, 25]. KRAS is therefore one of the important tumor pathogenic genes. Codons 12/13 in exon 2 of the KRAS gene lead to continuous activation of the EGFR signaling pathway that accelerates tumor cell proliferation. PCR in our case detected seven mutation hotspots at codon 12 and codon 13 in exon 2 of the KRAS gene (G12A, G12C, G12D, G12R, G12S, G12V, G13D), as well as mutation hotspots in exon 3 and exon 4 (Q61L, A146X). Our test results showed a G12D mutation in exon 2. In previous studies, 4 hotspot mutations were found in codon 12 in exon 2 (G12C, G12D, G12V, G12R), with an incidence of 80% to 90% [12, 17, 19, 26]. Other studies found that in the absence of the KRAS mutation, other somatic mutations detected by NGS in PRNRP included BRCA2, BRIP1, RAD50, TP53, and BRAF [26–28]. In cases of overlapping histology, immunohistochemical staining and KRAS mutational analysis can help make the correct diagnosis.

UC usually (90–95%) occurs in the bladder, but rarely (5–10%) occurs in the renal pelvis/ureter (i.e., UTUC). UC is more common in RTRs than in the general population. Genomic alterations may differ between Western and Chinese UC patients, especially differences in the genetic background and exposure to aristolochic acid in Chinese herbal medicines [29]. In addition, fewer drugs are developed and approved in China for advanced UC, with UTUC patients often suffering from chronic kidney disease, which makes them unsuitable for platinum treatment. It is therefore important to understand and develop therapy for the genomic characteristics of Chinese UTUC patients. In the present case, NGS was performed and detected the S249C mutation in exon 7 of FGFR3, a Q271Ter mutation in exon 10, and an A782Lfs mutation in exon 17 of KDM6A. In contrast to our test findings, Lai et al. [30] reported that there was no FGFR3 mutation in new UTUC patients after RT, which indicated that the mutation responsible for UTUC in patients after RT may be more complex. The FGFR3 gene encodes fibroblast growth factor receptor 3 and belongs to the family of tyrosine kinase receptors (FGFR1-4). The combination of FGFR3 protein with its cognate ligand fibroblast-growth factor (FGF), leads to receptor dimerization, which subsequently regulates cell proliferation, differentiation, migration, and apoptosis [31, 32]. FGFR3 exists as two isoforms, FGFR3b and FGFR3c. FGFR3b is the predominant form in epithelial cells and derived tumors. NIH-3T3 cells transfected with a mutated form of FGFR3b—FGFR3b-S249C induce cells to transform into spindle cells that have a higher proliferation rate and are tumorigenic in nude mice [33]. Activating point mutations in FGFR3 are observed in up to 70% of bladder cancers, with the S249C mutation being the most common point mutation, accounting for 69% of all mutations [34]. That study also found a relationship between FGFR3 mutations and the stage and grade of UC, with the frequency of FGFR3 mutations decreasing with increasing tumor stage and grade [35]. A recent study also showed that mutations in FGFR3 correlated strongly with the T-cell-depleted phenotype of UTUC. FGFR3 may remodel the immune environment of a UTUC by upregulating interferon response genes to reverse its T-cell-depleted phenotype. The authors of that study also proposed to use of FGFR3 inhibitors in combination with PD-1/PD-L1 inhibitors as a targeted therapeutic strategy to regulate the T-cell-depleted phenotype of UTUC [36]. Erdafitinib is a pan-FGFR tyrosine kinase inhibitor and the first FDA-approved targeted therapy for metastatic urothelial carcinoma (mUC) with FGFR2/3 alterations [37]. One case of advanced UC and mUC after RT has been reported with significant tumor regression and stable graft function after the administration of a combination of PD-1/PD-L1 inhibitors, chemotherapeutics, and immunosuppressants [38, 39]. KDM6A (lysine-specific demethylase 6A) encodes a chromatin-modifying enzyme that mediates transcriptional coactivation by functioning as a dimethylation and trimethylation histone H3 lysine 27 (H3K27) demethylase. Part of this effect is achieved by antagonizing histone lysine N-methyltransferase EZH2, while KDM6A-inactivating mutations may confer sensitivity to EZH2 inhibitors [40]. Compared with normal urothelial samples, the expression level of KDM6A in UTUC specimens was shown to be significantly reduced, while low KDM6A expression was associated significantly with higher tumor grade, shorter cancer-specific and disease-free survival time, suggesting that low expression and downregulation of KDM6A may accelerate the progression of UTUC [41]. Conversely, there is evidence that overexpression and upregulation of KDM6A are associated with poor prognosis in breast cancer and clear cell RCC [42–44]. Other studies also reported that mutations in FGFR3 and KDM6A were more common in low-grade UTUC [45–47] and were significantly associated with the risk of UTUC recurrence [48]. It is necessary to further investigate the function, pathogenic mechanism, and mutation status of FGFR3 and KDM6A. However, there is currently no consensus on the optimal therapeutic management of UC after transplantation. Physicians are cautious about using immunotherapy, given the transplantation rejection and the safety and efficacy of the new therapeutic regimen, such as checkpoint inhibition therapy or FGFR3 inhibitors. When a death occurs due to tumor progression, immunotherapy is not provided as first-line treatment for patients, but as a "remedial" therapy, which may have important value in clinical management strategies.

The potential mechanisms of cancer development after RT are complex, with the major mechanisms that may be involved including the use of immunosuppressive agents, decreased immune surveillance, and an oncogenic viral infection [49]. The application of immunosuppressants after RT puts the recipient in a state of immunosuppression for a long time. At this time, the cellular immune function of the recipient is severely suppressed, resulting in weakened or damaged immune surveillance function of the body, unable to remove cancerous cells in time. This leads to Viruses, including cancer-causing viruses, increasing the chances of infection and greatly increasing the incidence of tumors. According to animal experiments by Rovira et al [50], CsA down-regulates T-bet on the surface of CD8 + T cells, resulting in a decline in the immune surveillance of CD8 + T cells on tumor cells, thereby leading to tumor growth. Calcineurin inhibitors (CNIs), such as cyclosporin, raise transforming growth factor (TGF-β) levels which may promote tumor growth [51]. Jiyad et al [52] systematically analyzed that MMF’s carcinogenic effects. After RT, two types of tumors with distinct shapes occur in the autologous kidney, and the mechanism of occurrence is still unclear. Three inferences were put forward to explain its mechanism: (1) The two tumors may develop adjacently due to the presence of common oncogenic factors in this region, such as immunosuppressants, malignant transformation and changes in the microenvironment, etc. (2) A tumor stimulates the adjacent tissues of the tumor by secreting potential carcinogens, and induces the occurrence of adjacent tumors. (3) Gene mutation is also a potential cause. Network-based analysis of muscle-invasive bladder cancer (MIBC) samples by Kamoun et al. [53] revealed six categories: luminal papillary (LumP), luminal unspecified, luminal unstable (LumU), stroma-rich, basal/squamous (Ba/Sq) and neuroendocrine-like (NE-like). All luminal subtypes overexpressed markers of urothelial differentiation (FOXA1, GATA3, and PPARG). A study found that the luminal subtype had a higher frequency of KRAS mutations [54]. In this case, no KRAS mutation was detected in UC, but a KRAS mutation was detected in PRNRP. Interestingly, GATA3 expression was present in both UC and PRNRP. GATA3 is expressed in the epithelium of the bladder, ureters, renal pelvis, collecting ducts, distal tubules, and mesangial cells. GATA3 is a very sensitive and specific marker for urothelial carcinomas and their variants [55]. Tong et al. [19] confirmed that the tissue source of PRNRP is closer to that of the distal tubule epithelium using bioinformatics cluster analysis. The finding by Saleeb et al. [56] of enrichment of the nuclear receptor transcription signaling pathway in PRNRP, and GATA3 is a component of this signaling pathway. Studies have shown that overexpression of GATA3 was found in KRAS-driven lung cancer cells and further promoted tumorigenesis through microRNA [57]. Whether there are common gene mutations in the pathogenesis of UC and PRNRP, and whether there is an association between GATA3 and KRAS, needs further study.

## Conclusions

We report here for the first time an extraordinarily rare case of synchronous renal tumors of a PRNRP and UC in the ipsilateral kidney of an RTR. We identified simultaneous KRAS, FGFR3 and KDM6A mutations in the two different renal masses in the ipsilateral kidney. Pathologic assessment with comparative molecular analysis of mutational profiles facilitates tumor studies after RT and may be of great value in clinical management strategies.

## Data Availability

The relevant data and materials pertaining to this study are available upon request.

## Abbreviations

RTRs: Renal transplant recipients
PRNRP: Papillary renal neoplasm with reverse polarity
UC: Urothelial carcinoma
RT: Renal transplantation
CT: Computed tomography
PRCC: Papillary renal cell carcinoma
RCC: Renal cell carcinoma
IHC: Immunohistochemistry
PCR: Polymerase chain reaction
NGS: Next-generation sequencing
CsA: Cyclosporin A
MMF: Mycophenolate Mofetil
Pred: Prednisone
MSI: Microsatellite instability
UTUC: Upper urinary tract UC
mUC: Metastatic urothelial carcinoma
CNIs: Calcineurin inhibitors
MIBC: Muscle-invasive bladder cancer
